# Implementation as interaction of research, practice, and policy. Considerations from the Austrian initiative IMST

**DOI:** 10.1007/s11858-021-01300-y

**Published:** 2021-08-30

**Authors:** Konrad Krainer

**Affiliations:** University of Klagenfurt, Klagenfurt, Austria

## ‘Implementation’ as a challenge for different stakeholders

Koichu et al. (2019) regarded “implementation as a change-oriented process of endorsing an action plan”. In this view, implementation indicates an intended change in which relevant stakeholders notice a difference between a current situation and a desired situation (e.g., students’ better mathematical achievements). Based on this characterization, the “action plan” is the strategic instrument for improving the situation. Century and Cassata (2016) regarded implementation as being close to the idea of innovation, defining implementation research as “systematic inquiry regarding innovations enacted in controlled settings or in ordinary practice” (p. 170), stressing the importance of considering the particular (local) context. Implementations, innovations, interventions etc. are done in order to initiate a change, to make a difference.

In system theory (Willke, 2005), observation is regarded as noticing a “meaningful difference”, and intervention as “effecting” (generating, making) the “meaningful difference”, thus “implementing” steps towards the desired situation. Experts and laypeople differ in their art of observation: whereas experts are able to notice meaningful differences, problems, communalities, patterns, rules etc., laypeople are not able to do so (at the same level). This aspect raises several questions: Who are the relevant stakeholders (experts) who could/should decide what a “meaningful difference” is, and (based on that) if and when should a necessary change be aimed at (what problem should be solved)? Who is responsible for the implementation (for solving the problem, for initiating and disseminating the innovation, etc.) and who should decide whether the implementation has been successful?

Koichu and colleagues (2019) also indicated that implementation occurs in interaction of two communities, “a community of the resource proponents and a community of the resource adapters”. The “proposers” could be policymakers and the “adapters” teachers, but many more combinations are possible, including various roles researchers could take. Questions such as the following arise: Who are the relevant stakeholders whose voices should be heard when discussing implementation? What is the role of policymakers, administration experts, researchers and practitioners with regard to defining and solving problems that occur in practice?

In the following, four combinations of individual stakeholders are regarded as an example of a starting point for sketching the manifold contexts and challenges of implementation. In order to sketch the views of these stakeholders on implementation succinctly, the dimensions of concrete and general goals, and of short-term and long-term goals are considered, generating four combinations: concrete and short-term goals, concrete and long-term goals, general and long-term goals, and general and short-term goals. It would be easy to generate further examples and to substitute individual stakeholders according to teams, communities, associations etc., but even these examples should suffice to sketch the manifold challenges. The examples do not reflect real situations, but include elements of experiences and observations from various contexts.

### Concrete and short-term goals

Let’s assume there is a mathematics teacher called Anita. She teaches grade 6 students in several mathematics classes (with about 25 pupils in a class). Results of a questionnaire and a diagnostic test she asked her students to complete showed that both the motivation to learn mathematics and the abilities were very heterogenous. In order to offer good learning environments to all students, together with her colleague Alfred she started a 3-weeks project dealing with triangles (geometry), using sets of tasks with increasing cognitive demand (differentiation) and a flipped-classroom-approach in order to spend more time in class for joint discussions and individual support. They gathered the needed background information (questionnaire, diagnostic test, project plan, tasks on triangles, idea of differentiation, flipped-classroom-approach) from different sources (attendance of professional development courses in mathematics education, material at the ministry website and in a practice-oriented journal, hints from colleagues, and their own experiences). It was not easy for them to get material or support for their concrete project (within the short preparation time)—they found good examples of differentiation in mathematics teaching, but not dealing with triangles; they found good examples of project plans, but not focusing on flipped-classroom-approaches in mathematics, etc. The 3-week-project received a good evaluation from the students, and the two teachers learned a lot. However, also some questions regarding the value of their findings arose, and they became interested in results by professional researchers. Their new knowledge was not really spread to other colleagues, since there was neither a tradition of professional dialogue among mathematics teachers nor an established culture of exchanging innovations at this school or in the district (fostered by the principal or by administrative staff). Having such support, they might have contacted a regional education administration site or mathematics educators directly in order to get support as a group of mathematics teachers; they decided to wait for an opportunity for a collaboration, initiated by themselves or by external stakeholders.

Regarding implementation of research, it is evident that Anita and Alfred use information and sources that were partially generated and/or provided by researchers, probably accompanied by an action plan. One can say that they implemented research-based sources, however, not as part of a running implementation process. Due to the lack of a professional community at their school and in the region, the bridge to research was not yet existing.

### Concrete and long-term goals

Barbara is an experienced mathematics educator and researcher, specialized in arithmetic and algebra education at the secondary level. Together with her doctoral student Belinda, she constructed a diagnostic instrument for students’ knowledge about fractions in grade 6. In order to use the instrument for improving mathematics teaching regarding fractions and carrying out corresponding research, they generated a professional development course that had a double goal: on the one hand, it should help 20 teachers to understand and use the instrument in their grade 6 classes, contributing to an improvement of students’ thinking (knowledge, beliefs); on the other hand, they wanted to investigate the impact of the professional development course, also compared to a course with another 20 teachers where an intervention not using the instrument was carried out (control group). The project received a good evaluation from the teachers, (not surprisingly) especially from those working with the diagnostic instrument. These teachers improved their knowledge, many of them changed their teaching in the direction of more student-centeredness, and some of them even became more interested in research and got involved in further studies of the researchers. The researchers further improved their instrument and published their results in high-impact journals, indicating the successful implementation of their professional development course, heavily based on the instrument. They indicated that it would be interesting to scale up their interventions and related research. Most of the 20 teachers working with the diagnostic instrument had similar views: they worked at 10 different schools and realized that it would be beneficial to have this practice-research-collaboration impacting all mathematics teachers at their school, not only two of them. The participating teachers themselves did not feel competent enough to spread their new knowledge to others; they felt more like learners than teacher educators. The principals spoke with the regional education administration site regarding a scaling up process, but so far, the negotiations between the policy and the research communities have no breakthrough. Among others, challenges refer to the fact that Barbara would further be interested in combining the professional development with research, needing additional research assistants, among others substituting Belinda who would be leaving the university after obtaining her Ph.D. Working with 100 teachers would be the maximum that Barbara could manage, implementing her approach with the same intensity as in the pilot. In contrast, the policymakers wanted to reach considerably more people in a shorter period of time, and aimed at minimizing the costs for scaling up. Some of the 20 teachers—when asked by the policymakers whether they would eventually take a multiplier role—articulated interest, but also demanded that they should be provided with an additional course, in particular indicating the need for advisory competences and a kind of mentoring by Barbara. Also, a question arose which incentives the teachers should get for their additional work beyond their main work as teachers. So far, the negotiations continue, and a matching of the different interests among practice, research, and policy is not yet in sight.

Regarding implementation of research, the project showed many kinds of positive aspects regarding practice, research, and policy. The challenge is the missing scaling up in each of these communities. Hindering factors are the small number of researchers who feel able and ready to be involved in larger intervention studies. The issue of promoting or even establishing expert teachers needs to be considered. Overall, budget considerations play a limiting factor.

### General and long-term goals

Carlo is an educational researcher dealing with the interplay between instructional and school development and is a renowned expert in quantitative methods. Although he has a background of a few years of mathematics teaching, his research interest is not focused on mathematics teaching, but is more general regarding all subjects, and in particular interdisciplinary and project work. When Chris, a representative of the ministry, contacted Carlo to think about a plan to bring about change in mathematics teaching in the country in order to react to recent findings of an international achievement study, they met for a brainstorming session. The basic idea by Chris was working with 1000 (primary and secondary) schools improving their scores regarding the educational standards in mathematics and reading within three years. Carlo was concerned by the large number and the time. He suggested to start first with a pilot project with 20 schools, considering the impact not only on mathematics and reading, but on all subjects, involving the whole staff of the schools in order to initiate a new culture of learning at these schools, indicating the pivotal role of principals. Chris, although not totally convinced concerning this idea of using a slow process and comprising only a small number of schools, asked Carlo for an implementation plan, because he felt much pressure to present a plan to the minister who in turn felt a need for reactions to media reports about the study results. One challenge facing Carlo was the fact that he needed experts from subject didactics, in particular mathematics education since a necessary link to educational standards was expected. The task was complex, since the experts in the different subject didactics were few in number, but also possibly had very different views on teaching and the direction of changing teaching. Also, the methodological expertise of the colleagues that Carlo contacted differed greatly, as did their readiness and views of providing professional development courses. In sum, it seemed not to be easy to get all experts under one umbrella regarding the intervention (regarding the question of what is the major focus of change) and the related research (which effects are most interesting?). When Carlo and Chris met for another meeting, Chris reported about an intervention initiated by a teacher union, in which they heard about the ‘huge intervention’ (by policy and research) and asked for an active involvement of practitioners from the beginning in order to avoid unforeseen problems when implementing the idea. A group of mathematics educators wrote to the ministry that they principally supported such a project but that it should be guaranteed that it showed a clear focus on mathematics, in order to support students’ mathematical thinking and not ending up in general discussions. Negotiations are ongoing.

Regarding the implementation of research, the initiative raises the question of quick scaling up and seems to have an adequate social entity involving schools in order to promote changes. The challenges here are the undefined intersection between concrete (subject-specific) students’ learning and organizational learning, and a process of integrating practitioners’ view into the implementation, reflecting future developments at the schools, in particular the steps needed after the researchers and professional development providers have left.

### General and short-term goals

Drake, working in the ministry with a profound background in management, took part in a certificate ceremony where Deborah, a mathematics teacher graduating from a 2-year masters course for expert teachers sketched the idea of educating multipliers who themselves provide courses for other teachers. Drake saw a lot of potential in this idea: one starts with 20 teacher educators, each of them providing an expert teacher qualification (ETQ) course for twenty teachers (within some months); again, each of these (new) expert teachers provides an ETQ-course for again 20 teachers; this leads to 8000 expert teachers for mathematics within a year, and similar things could be done for other subjects. In order to bundle synergies, expert teachers from different subjects can work together at one school to push learning forward. Deborah, who learned much in the graduate course about constructivist teaching and the challenge of transforming knowledge, saw some benefit in thinking about scaling up expert teachers like her, but articulated also a lot of concerns: even if it works finding twenty mathematics educators, etc., it would not be easy for them to implement the idea in a similar format; and even if this would be more or less given, the next two courses provided not by them—but by graduates and graduates of graduates—could lead to very different effects, as games like ‘whisper down the lane’ show; also, she doubts whether this could be done in such a quick time and whether teachers would accept ‘third generation’ expert teachers. It was not easy for Drake to see the limits of his multiplier-approach (easily reaching 20, 400, 8000, …); he indicated that he wanted to think further about that, inviting other experts to check alternative models. Regarding the implementation of research, the dialogue between the two representatives of policy and practice shows that the question of scaling up—from a quantitative point of view and assuming that a country has enough qualified teacher educators interested in intervention research projects commissioned by policy—is not that demanding. The challenges are time, quality, collaboration, and—as a consequence—also budget.

The four examples show that the challenges of implementation are manifold. They demonstrate that the implementation of research, and its implementability, are dependent not only on researchers and on practitioners, but also on policymakers, too, in particular if scaling up is regarded as important (e.g., Adler et al., 2005). The examples indicate various types of intersections and collaborations among the three communities *practice*, *research,* and *policy* (including steering-relevant administration), also making opportunities and limitations visible. It makes plain once more, regarding research aiming at contributing to improving teaching, that teachers and—to some extent also administrators—need to be seen as stakeholders.

After this heuristic introduction, theoretical considerations have the potential to sharpen the issue. In the following, two contrasting approaches related to *implementation* and *implementability* of research are sketched and analyzed, namely ‘technical rationality’ and ‘reflective rationality’.

## From ‘technical rationality’ and ‘reflective rationality’ to ‘societal rationality’

Schön (1983) introduced the term *technical rationality* into the educational discourse. It follows three basic assumptions (for a more extensive discussion with regard to the teaching profession see Altrichter et al. 2008, p. 270):

- There are general solutions to practical problems.
- These solutions can be developed outside practical situations (in research or administrative centres).
- The solutions can be translated into practitioners’ actions by means of publications, training, administrative orders, etc.

In contrast to technical rationality, *reflective rationality* (e.g., Altrichter et al., 2008, p. 270), building on the notion of “reflective practitioner” (Schön, 1983), follows three very different assumptions:

- Complex practical problems require particular solutions.
- These solutions can be developed only inside the context in which the problem arises and in which the practitioner is a crucial and determining element.
- The solutions can only rarely be successfully applied to other contexts, but they can be made accessible to other practitioners as hypotheses to be tested in practice.

It is evident that these two approaches are to some extent constructed to indicate a stark contrast. Whereas *technical rationality* starts its ‘rational’ from the view of externals looking at practice, *reflective rationality* starts its ‘rational’ from the view of the internal experts. The different starting points could lead to ‘meaningful differences’ regarding views on implementation, but not necessarily. In the following, strengths and weaknesses of these two approaches are reflected. This contrast might help to sift out the ‘meaningful differences’ of the two approaches and eventually lead to regarding the differences as a chance to go beyond only viewing differences.

### Technical rationality

The major strength lies in the fact that externals can look at commonalities of different action constellations and their elements (which requires comparisons that suggest an external perspective). The more context factors can be kept constant, the more it is possible to generate general knowledge or regulations. Based on this strength, stakeholders outside practice (researchers, educational policymakers, etc.) are explicitly regarded as co-responsible for dealing with challenges in educational practice. More concretely, research and administrative centres are expected to contribute to defining, solving, and disseminating solutions.

The major weakness is the low level of context sensitivity that is closely related to this strength. When context factors play a pivotal role (each class is different due to different students, the teacher, the school context etc.), *technical rationality* naturally reaches its limits more quickly than in areas of phenomena that are less context-sensitive. Assuming that context factors can be kept constant, practitioners are not explicitly regarded as being responsible for co-defining, co-solving, and co-disseminating solutions. This could be read that practitioners could/should only put into practice what externals determine. This position underestimates the local experience and knowledge of practitioners and expresses a rather strong belief in a hierarchy of knowledge. The attribute ‘general’ in *general solutions* weakens this approach since individual practitioners would hardly expect that research would find general solutions for dealing with particular (mathematical or pedagogical) problems.

### Reflective rationality

The major strength is its sensitivity to context. The knowledge gained helps participants to cope with situations, considering the specific context conditions that are accessible only to the practitioner who acts in this situation. Based on this strength, practitioners are regarded as fully responsible and capable of dealing with challenges in their practice. They are expected to contribute to defining, solving and disseminating solutions. The approach indicates the importance of *particular* solutions to complex practical problems. Related to that, the term ‘contexts’ indicates the importance of a situated (context-specific, non-general) view on problems and solutions, addressing the challenge of applying situated solutions to other contexts, thus highlighting the delicate issue of scaling up solutions (innovations, regulations, etc.).

The major weakness is that the knowledge gained in the process cannot be simply generalized (a similar discussion relates to the question whether case studies can be generalized, e.g., Hammersley et al., 2000). Although practitioners’ knowledge can be conveyed to other practitioners as individual experiences, such knowledge has to be checked again in their context and usually also modified. Assuming that general solutions are not possible, neither researchers nor other externals are mentioned as stakeholders in this approach. Thus, such experts are not explicitly regarded as co-responsible to co-define, co-solve, and co-disseminate solutions in practice, or to play a supportive role (via observation, feedback, input etc.). This could be read as underestimating the role of research and administrative work and as a rather strong belief in total situatedness of knowledge.

### Comparison of strengths and weaknesses of two approaches

What are the ‘meaningful differences’ between these two approaches? The key terms are ‘general’ (knowledge) and ‘context’ (sensitivity). The more complex a context is, the more factors have to be considered when solving a problem; the more sensitivity is needed to see the *particularity* (uniqueness, genuinity, concreteness etc.) of the problem, the less easy is it to see solutions as *general*. It seems that the meaningful difference lies between the *general* and the *particular*. It is not wise to generalize *particular* solutions, but it could be a good starting point for finding *particular* solutions in another context. It is not wise to believe in *general* solutions in a general way; however, it makes sense to reflect on important context factors and to find (relative) general solutions to problems, keeping some (rationally) selected context factors constant. This knowledge can be used as a starting point for solutions in other contexts, in particular, when similar context factors are given. Thus, there is no need to see the *general* and the *particular* as opposite, but as complementing each other. One can always search for the particular in the general, or the general in the particular.

The four examples in Sect. 1 show that it makes a difference whether an initiative focuses on a very concrete (specific) topic such as triangles or fractions (at specific age levels, examples A & B), or whether changes in mathematics teaching in general (eventually at many or even all grade levels, examples C & D) are intended (or even a change of teaching in all subjects). Also, it makes a difference whether an initiative is intended to reach long-term goals (examples B & C), or whether it aims at reaching goals within three weeks or within a year (examples A & D), whereby the intended initiative’s complexity in example D (quick change in a short time, with a very general and ambitious goal) reveals fundamental tensions.

How does this relate to the work of practitioners, administrators, and researchers? In general, *practitioners* are more interested in solving their particular (local, concrete etc.) problems, but they can learn a lot from more general (global, theoretical etc.) views. The nearness to one’s own practice can be an advantage (having more situated knowledge than externals), but at the same time a disadvantage (having less distance to one’s actions, being trapped in one’s views on problems and solutions). In general, *administrators,* such as ministry representatives, are more interested in general solutions, not being able to think about each particular school, teacher, or even student. Therefore, they need and like numbers when trying to understand problems and possible solutions. However, administrators also love concrete examples and stories in order to understand problems and solutions (numbers and stories representing the general and the particular). *Researchers* need to believe in both, the general and the particular. For example, in teacher education we can do a study of 1000 mathematics teachers’ beliefs about infinity and a study on one single teacher’s practice over five years. Both, working with large numbers (quantitative research), with particular contexts (qualitative research) or mixing them, depends on the research question. Meta-studies regarding teacher education research show that small-scale studies (N < 20) and qualitative approaches dominate (e.g., Adler et al., 2005; Gellert et al., 2012; Krainer et al., 2021). The reason could be that the field of mathematics teacher education research is still emerging, but it could also be the (multi-factorial) context that makes teacher education research more attractive to looking for the particular. Anyway, if scaling up is an issue for improving teaching in a whole country, and is focused not only on some teachers in a research study, then *implementation* as an issue becomes more important.

Implementation of research cannot be viewed only as a quantitative problem of finding general solutions. The plan can be a general one, but the act of implementation is a particular act by each practitioner. Implementation needs to be seen as a process in which the *general* and the *particular* are to be reflected and to be brought into a certain balance, influenced by the complexity of the problem, and in particular by the quantity of people involved. If research results do not impart a meaning to practitioners that extend their existing views, the results will have no strong impact on their knowledge, beliefs, and practices. If practitioners reflect only on their own specific context, they will not easily be confronted with alternative, provoking, theoretical, general, etc., views from research (but also from research-informed policy), motivating them to (partially) further develop their knowledge, beliefs, and practices. Thus, *technical rationality* (focusing on *generalization*) and *reflective rationality* (focusing on *particularization*) in their idealistic forms need to be connected, regarded at a higher level not as opposite, but as complementary.

Each implementation that aims at spreading to a larger number of people needs to include the perspectives of practice, research, and policy. Thus, the process has a *societal* dimension, involving different stakeholders, jointly being co-responsible for a successful implementation. Therefore, a third approach, building on the strengths of the two mentioned approaches, is developed below.

### Societal rationality

This approach follows three assumptions:

- Practical problems require an adequate link between general and particular solutions. The more complex the problem, the more important the particular.
- The solutions gain in quality if all concerned (including policy, research, and practice) are involved in the problem definition and in the solution and evaluation process.
- The solutions can at best be partially applied to other contexts. Concrete examples, critical reflections, theoretical considerations, empirical findings, general guidelines, specific or general quality criteria can be used to adapt solutions context-sensitive.

The sketched initiatives in the four examples in Sect. 1 could profit from reflecting where *technical* and *reflective rationality* views seemingly get into conflict. It might be possible to understand and possibly overcome some tensions, proceeding towards a *societal rationality* approach.

In the following, the context of the Austrian long-term initiative IMST is used to reflect selected research and development activities. The reasons for the choice are as follows: it is long-term, involves practice, research, and policy, scales up innovations in MINT teaching (mathematics, computer science, science, and technology; partially also other subjects, see Sect. 3), and builds to a large extent on a bottom-up approach (near to *reflective rationality*) with some elements of a top–down approach (near to *technical rationality*). The reflection is done in chronological order, where different phases of implementation are described. It was the challenge of writing this paper that led to formulating the *societal rationality* approach as a third perspective, although never explicitly used before.

## On the implementation of the Austrian initiative IMST

As in other countries, *TIMSS 1995* evoked dynamic developments in the Austrian education system. Among others, it led to a *research project*, followed by further phases such as the *initiation of a national support system* and the *implementation of the support system*, and recently to *considerations about implementing the initiative* in a sustainable way.

### TIMSS 1995 as impulse for the initiative

Whereas Austria’s results concerning primary and middle school students’ achievements were rather promising, the results at the secondary level were disappointing. In particular, the TIMSS advanced mathematics and physics achievement test generated irritation: the ranking lists showed Austria as the last (in advanced mathematics) and the last but one (in advanced physics) among 16 participating nations (e.g., Mullis, Martin, Beaton, Gonzalez, Kelly, & Smith, 1998, pp. 129 & 189). These results—together with others—evoked a public discussion on educational practice, research and policy and indicated that the teaching of mathematics and science in Austria needed a shift.

TIMSS is an implementation aiming at new insights into the intended, implemented, and achieved curriculum at an internationally comparable level (marking a meaningful difference compared to former research knowledge). It was mainly stimulated as an interaction of two communities, namely *research* (commissioned by many countries, carried out by a large network of researchers and test centers) and *policy* (commissioning and co-financing TIMSS). Although the action plan for a study like TIMSS is not the task of *practice*, potential outcomes and decisions might evoke change-oriented processes and action plans with strong consequences for practice. Whether a study like TIMSS evokes any development in a country depends largely on the specific results of that country, and its interpretation of the results.

### A research project as start for a reaction to TIMSS (1998–1999)

The Austrian Ministry of Education (in the following, in brief called the ministry) commissioned a research team working out suggestions for improving the situation. This gave birth to the research project IMST (Innovations in Mathematics and Science Teaching, 1998–1999; e.g., Krainer, 2003). The findings (among others, using results of questionnaire and interview surveys with teachers, expert teachers, principals, teacher educators, regional and national policy representatives) revealed several challenges, in particular the following:

- Learning outcomes (e.g., poor achievements regarding higher levels of thinking);
- Teaching processes (e.g., fewer students in Austria than in most other countries were well involved in reasoning tasks);
- Status of mathematics and science education (e.g., only at some universities there were professors and doctoral students within these disciplines, and none at other teacher education institutions);
- Situation of teacher education (e.g., weak exchange between primary and secondary teacher education);
- Instructional and school development (in particular, due to the last two issues mentioned above, there is a lack of a systematic support system for teachers and schools).

The analysis of data showed a picture of a fragmentary educational system with a consistent pattern regarding the state of mathematics and science teaching, teacher education and corresponding research in Austria: there was a lot of autonomy and action, however, little evidence of reflection and networking (e.g., Krainer, 2003). Thus, the main message of the research results was a systemic one: not only teachers needed to learn, but change was needed in the whole educational system, including principals, teacher educators, researchers, administrators, policymakers, and their corresponding institutions such as schools, teacher education and research institutions, administration and policy bodies including the ministry, as well as the society itself.

The IMST research project is an implementation aiming at new insights into the situation of mathematics and science teaching and teacher education in Austria (marking a meaningful difference from former knowledge). Also, this project was mainly stimulated as an interaction of *research* (commissioned by the ministry, carried out by a network of mathematics and science education researchers) and *policy* (commissioning and financing the project). However, teachers, superintendents, teacher educators and other experts from *practice*, policy, and research were partially involved in data gathering and in feedback loops where findings were presented and discussed. The idea behind this approach was to use parts of data gathering as a process of interest-building by people from practice, policy, and research, and of facilitating a potential following implementation in practice (and partially also in policy and research). Thus, the action plan for the project was not the task of practice, but through involving practitioners it facilitated its endorsement through relevant parts of practice in Austria and prepared the field for innovations. Although not named as such, the genesis of IMST is one closely related to a *societal rationality* approach, regarding communities of research, policy, and practice as important groups to bring about change. The ministry appreciated the efforts by the communities of research and practice; however, it never aimed at branding the initiative as a ‘reform project’ (probably because it would have indicated weaknesses in the education system and a strong need to overcome them).

### The initiation of a national support system (2000–summer 2004)

IMST was further commissioned by the ministry for the period 2000–2004 with the aim of fostering innovations at secondary schools, and of proposing a long-term plan for improving the situation. In order to take systemic steps to overcome the fragmentary educational system, the approach of a ‘learning system’ (e.g., Krainer, 2002, p. 26) was taken. A major assumption was that the dimensions action and reflection as well as autonomy and networking needed to be kept in a certain balance, whereby—due to the dominance of autonomy and action—the promotion of reflection and networking was regarded as the major intervention strategy. The concept of a *learning system* makes use of several theoretical backgrounds, including action research, constructivism, network theory, system theory, and community of practice (see e.g., Krainer, 2002). IMST regards students, teachers, teacher educators, and administrators as inquiry-based learners (e.g., Krainer & Zehetmeier, 2013). In particular, teachers are seen as experts who investigate their own teaching in a systematic and self-critical way (action research, e.g., Altrichter et al., 2008). They are key agents of change getting support from policy and research in the sense of a *societal rationality* approach.

The proposed long-term plan was to establish (starting with the school-year 2004/2005) a nation-wide support system for mathematics and science teaching in Austria (Projekt IMST^2^, 2003). A particular focus was laid on evaluation and research, and on gender and diversity, which should be integrated into all measures.

The major measures were as follows (see more detailed in Krainer, 2015):

- *Multiplier and interface structures*: Establishing a subject-related middle management at the local and the regional level (at schools and in the federal states) who could facilitate implementation through supporting teachers in disseminating innovations and building bridges between policy, research, and practice.
- *Qualification and research structures:* Establishing Austrian Educational Competence Centres (AECC) and regional subject didactics centres, in order to have an adequate academic basis for doing research in subject didactics, for (further) educating prospective and practicing teachers, as well as implementation facilitators.
- *Support structures for practice:* Establishing a fund where teachers can submit project proposals, in order to make good practices visible and accessible to all teachers, and in order to use them in teacher education and research.
- *Network structures:* Establishing Regional Networks in all nine federal states in Austria, in order to broaden the initiative at the regional level and to generate innovations specific to the different contexts in the federal states.

Regarding implementation, this means that the action plan involves *policy*, *research,* and *practice*, however, with different roles: the policymakers (ministry) commissioned the plan and agreed to support its implementation as far as the budget and the policy of the respective government allowed; the research community submitted the plan and advised the ministry regarding the necessary decisions (IMST team) and contributed to implementing the structures and to evaluating and investigating it (various members from universities and other partners); the practitioners were expected to use the offered support structures (e.g., initiating and spreading innovations), and in particular expert teachers were motivated to offer their expertise regarding all four structures mentioned above, or to attend professional development programmes or other learning opportunities qualifying them to act as brokers between the communities of practice, research, and policy.

The action plan was ambitious, with long-term goals, both general (at the national level) and concrete (at the teachers’ level). Since the intervention intended that not only teachers need to learn, but the whole educational system, the plan meant following a *societal rationality* approach.

### The implementation of the support system and related changes (autumn 2004–2018)

Although the period of time is long, the course of the initiative was and still is rather full of challenges, changes and adaptions. In each of the five 3-year contracts (2004–2006 till 2016–2018), new issues had to be negotiated, partially due to changes of governments (with different political parties involved), changes of ministers (sometimes representing education and research, sometimes representing other combinations such as education and women, or economy, science and research), new institutions (e.g., the foundation of “Pädagogische Hochschulen”—university colleges of teacher education—in 2007) or new reforms (e.g., the legal anchoring of educational standards in 2009).

By the end of 2018, the *status of implementation* of the major measures regarding the three communities—policy, research, and practice—was as follows:

- *Multiplier and interface structures*: With the exception of a 2-years pilot programme with about 90 expert teachers graduating, no corresponding programmes were implemented. These structures would have been the strategic pool of expert teachers building bridges to policy and research. Only in a few federal states, a subject-related middle management at the regional level was implemented, and several graduates of the pilot programme made careers in administration or at universities or university colleges of education. The hoped bridge-building function between the three communities could not be reached.
- *Qualification and research structures*: During the years 2004–2006, six AECC had been established (among others, one for mathematics education). They initiated research projects and doctoral programmes, contributed to the generation of educational standards, the standardized final examination at the end of secondary schooling, and the new teacher education. Such a bridge-building between research, policy, and practice was also done by more than twenty newly established regional subject didactics centres, some of which became Regional Educational Competence Centres (RECC). All these centres contributed largely to the further development of the disciplines. The resources came both from the ministry (policy) and from the corresponding universities or university colleges of education (research). New positions were established, with career chances both for young researchers and expert teachers from schools, partially also for policymaking.
- *Support structures for practice*: IMST established a kind of fund, structured in programmes dedicated to challenging topics (e.g., learning with digital media), led by a team of experts from research and practice. Each programme supported about 20 innovation projects a year all over Austria. The reports of the teachers were published on the IMST-Wiki (http://www.imst.ac.at/wiki) website. Selected projects were presented at IMST conferences, network meetings, partially at international conferences and in the context of EU-projects (e.g., Fibonacci, KeyCoMath, and PROFILES, PARRISE). These *structures* showed an intensive collaboration between the research and practice communities, since many support activities were done by teams of researchers and teacher experts. The policy was involved in discussing the topics, the evaluations, and the further development of the programmes. The jury of the IMST award for excellent projects consisted of experts from research, practice, policy, and economy.
- *Network structures*: Till 2008, Regional Networks were established in all federal states. Since then, these contracts (e.g., including the fact that the federal state invests more resources than provided by IMST) have been prolonged in all cases till the end of the period. They established platforms for schools and teachers, setting up opportunities for sharing experiences and further education, supporting school development and small projects (which could lead to submissions in the fund), developing a pool of experts (e.g., Rauch, 2013). Often, the networks were the driving force for establishing regional centres for subject didactics or district networks. The Regional Networks are places where representatives of all three communities are involved, based on contracts with the regional boards and IMST. Biennial national meetings bring researchers and practitioners together, partially involving representatives of national and regional policy.

Looking back, the continuous involvement of all three communities—in the sense of a *societal rationality*—seemed to be a major factor for the long life of IMST. That not all measures have been implemented in the intended way, seems to be a real life-experience when working at a national level where political decisions are needed. Many members contributing to IMST as a network of individuals, teams, organizations, etc., were surprised that any suggested measures were implemented at all; in particular, the establishment of AECC was regarded as something not expected at all. On the one hand, IMST (originally focusing only at the upper secondary level) was extended to all school levels and types, and subjects like German language were added (which was the main reason to rename IMST into Innovations Make Schools Top); on the other hand, stagnating resources (due to state-wide budget problems) and an increasing number of generic (not MINT-focused) reforms (educational standards, standardized final national exam, new teacher education, new middle school, school autonomy etc.) decreased the focus on subject-didactic, (in particular MINT), influencing the potential of IMST.

Whereas in the last decades of the twentieth century, the Austrian educational policy had been often criticized due to a lack of (national) reforms, starting in the late 2000s, a variety of reforms were launched. This dynamic was accompanied by a stronger emphasis on central (national) steering and by an increasing tendency of educational policy to favor a *technical rationality* approach. This tendency, together with budget problems and having no severe problems with achievements in MINT teaching, seem to be the main reasons for a decreased attention in policy to the (bottom–up-near) initiative IMST.

The achievement issue is a delicate one. Pretty much at the same time as the ministry implemented a law reducing the number of lessons for students at all Austrian school levels in 2003 (including MINT subjects), IMST started to support schools at the lower secondary level in 2004, and in 2007 at the primary level. The initiative’s representatives argued—in collaboration with practitioners and researchers—against the (long-lasting structural) reduction, however, without success. Nevertheless, the ministry supported the work of IMST, and thus, despite the feeling of loss, the challenge was to generate a dynamic in MINT teaching in Austria, and hopefully to make a contribution so that Austria does not fall back too much in international comparisons. A comparison of Austrian mathematics students’ achievements at the primary level (TIMSS 2007–2019) and at the secondary level (PISA 2003–2018) with the neighboring countries Czech Republic, Germany, Hungary, Italy, and Slovakia (Krainer, 2021) shows that regarding *significant differences* between Austria and the other five countries, Austria improved—at the primary level even considerably (e.g., overtaking Germany). Given the reduction mentioned above, the improvement of Austria is rather surprising. Recently, the international comparisons are not a really big issue in Austria, the focus is more on schools performing below their expectations regarding educational standards in mathematics and the German language at the primary level, with the addition of English language at the secondary level.

### Considerations about implementing the initiative in a sustainable way (2019–2021)

In the years 2019–2020, a sustainable anchoring of IMST as a collaboration of universities and university colleges of teacher education was anticipated to start in 2021. However, COVID-19 and open questions regarding the governance of IMST prolonged the negotiation into the year 2021). First, an action plan (Krainer et al., 2019) has been worked out to further develop IMST into a nationwide professional development system for schools (where individual ECTS credits can be collected and transferred into credits for the corresponding schools, leading to a potential accreditation as a LECC, Local Educational Competence Centre). Although supported by a large network of researchers, practitioners and further stakeholders (including some policy representatives), the action plan was not supported by some university teacher colleges of education and their responsible administration unit in the ministry, indicating the leadership of these colleges in the areas of professional and school development. They intended that IMST should be transferred into the responsibility of university teacher colleges of education. The IMST team, always regarding the initiative as a collaboration between universities, colleges, and further stakeholders, did not accept the exclusion of universities. As a compromise, a think tank (with representatives of ministry, practice, university, and university teacher colleges of education) was established to work out a joint plan. The think tank agreed on a new, rather open action plan in early 2021, a decision the ministry has not endorsed so far.

This means that the existence of two action plans—based on extensive negotiations, and involving policy, research, and practice—does not necessarily imply that an *implementation* has been decided upon. The main reason for not implementing such plans is that not content-related issues (MINT) are in the focus, but policy-driven issues (organizational power). Although establishing university teacher colleges of education and later establishing a responsible administration unit in the ministry for these colleges brought content-related advantages to the field, there were also disadvantages. Having different responsible administration units in the ministry for schools, university teacher colleges of education, and universities (including universities of applied sciences), led in the education sector to more competition between universities and colleges. The fact that the colleges are established as subordinate agencies of the ministry, and universities have more autonomy, makes a difference for policy when articulating demands (requiring as few resources as possible). Thus, the research community is somewhat split into interest groups, mirrored by corresponding administration units in the ministry, not fully considering the content-related (MINT) needs of practice and society. From a long-term point of view, a clearer focus on MINT (recently additionally forced by pandemic and climate challenges) would be needed in Austria. However, the growing tendency towards *technical rationality* makes implementing an initiative with a *societal rationality* approach—including much trust in *reflective rationality* and schools’ and teachers’ autonomy and professionalism—delicate.

Whereas, so far, the implementation of IMST has been reflected, a brief focus on implementing IMST *research* will follow now.

## On the implementation of IMST research

Research within the IMST initiative takes place regarding various contexts and topics:

- *Action research by teachers.* The IMST-Wiki is an internet platform with over 2100 good practice articles (reports on innovations carried out, reflective papers in the context of university courses etc.). Over 1600 of these contributions stem from the IMST initiative. Some of these teachers’ writings are the subject of case or cross-case analyses.
- *Research on particular aspects of action research.* Due to the emphasis of IMST on urging teachers to collect data, to reflect on their practice, to write down their experiences as a second cycle of reflection (and becoming public to others via the platform), we were interested to which extent the writing process is valued by teachers. In her dissertation, Schuster (2008) investigated teachers’ views on writing from different perspectives.
- *Research on sustainable impact of IMST.* When teachers participate in initiatives like IMST, changes in knowledge, beliefs, and practice occur. But do they last when participation ends, when external support is over? The empirical investigation of sustainable effects of IMST is the subject of the dissertation, habilitation and further papers by Zehetmeier (e.g., 2015), differentiating intended and not intended effects, and sifting out factors that promote or hinder sustainable effects.
- *Research on the scaling-up dimension of IMST.* The initiative aims at disseminating innovations, scaling up the number of teachers carrying out innovations at their school. A project compared new projects and follow-up-projects in mathematics regarding their degree of spreading innovations outside teachers’ own classrooms, or even beyond their own school’s (partial study in Krainer et al., 2018).
- *Basic research using data from IMST.* Hanfstingl et al. (2010) used data from student and teacher questionnaires to focus on the mediating role of teachers’ person-related variables between perceived psychological basic needs and intrinsic motivation to teach. Further, they investigated whether specific personality aspects are co-responsible for the level of intrinsic motivation.

This generated research knowledge flows through initial and further education, lectures and publications (science-to-science, science-to-professionals, science-to-public) in educational policy, educational research, and educational practice as well as (again) in IMST. A mediating role between the IMST-Wiki (teachers’ reflective papers) and contribution to the scientific community (research papers) is played by the so-called IMST-Newsletter, where teams of researchers and practitioners write (practice-oriented but research-based) special issues on specific topics, with “Distance learning in the time of pandemic: examples from teaching, teacher education and research” (Zuliani, 2021) and “Inquiry-based learning” (Koliander & Knechtl, 2020) as two recent ones. Theoretical and practical reflections on the subject of ‘knowledge transfer’ and ‘implementation’ stem from Krainer and Posch (2000), and Krainer and Zehetmeier (2013).

In a meta-study, based on five sub-studies on IMST, Krainer and colleagues (2018) examined promoting and hindering factors as well as challenges of disseminating innovations. Among other things, the establishment of a connection between individual and organizational learning and the balance between a bottom-up and a top-down strategy were identified as essential. This result supports the plea for subject-related education management (e.g., expert teachers), which was proposed as a major measure by IMST in the early 2000s, but which was implemented only partly (see Sect. 3). However, the results also contributed to the above-mentioned future action plan for IMST (Krainer et al., 2019) where this idea is taken up again, in particular focusing on the pivotal role of principals. If extended autonomy of schools is aimed at (which in Austria is intended, coupled with more accountability), then principals and subject-related education managers might help in fostering educational innovations and subject-related collaboration among teachers on scale.

## Lessons learnt from IMST on implementation

The reflection on IMST supports the approach of Koichu et al. (2019) in defining “implementation as a change-oriented process of endorsing an action plan”. The experience of the nationwide initiative IMST suggests that, in considering larger initiatives, *policy*, *research,* and *practice* need to be regarded as influential and closely interrelated communities regarding implementation. Also, the view of Century and Cassata (2016), who regarded implementation as being close to the idea of innovation and stressed the importance of considering the particular (local) context can be supported.

Reflecting the contrast between *technical rationality* and *reflective rationality*, in particular working out advantages and disadvantages, led to a specific insight: implementation of research needs to be seen as a process where the *general* and the *particular* are to be considered and to be brought into a certain balance, influenced by the complexity of the problem. Complex problems where implementation is aimed at spreading to a larger number of people need to include the views of those in practice, research, and policy communities, indicating the societal dimension of the process. Based on these considerations, a third approach, *societal rationality*, was defined.

The several phases of IMST (research project; piloting, planning, and implementing a support system) were carried out in the sense of a *societal rationality*. Research, practice, and policy were driven by the common view that—due to TIMSS (and later PISA)—the country has an urgent need to react to content-specific challenges. In the early 2000s, Austria had a lack of reforms and data about teaching and teacher education, the dominant steering approach was input-oriented. Teacher education was split into two institutions, with only universities having considerable research strengths, however, not enough in subject-didactics. There was no real competition between teacher education institutions and only a weak exchange between primary and secondary teacher education. The non-university teacher education institutions and the school boards in the nine federal states were mostly regionally steered with a rather surprising low national steering.

This situation changed gradually when, as in many other countries, the dominant steering approach became output-oriented. In trying to overcome a long period of lack of reforms, a variety of reforms were initiated in the late 2000s, with the tendency to strengthen central steering. Among others, educational standards and a standardized final examination were launched at a national level, new and more centrally steered school boards, and the new middle-schools (formerly main schools) were established. The non-university teacher education institutions were upgraded to university colleges for teacher education, centrally steered by a new established administrative unit in the ministry. All these reforms had rather a general and not a subject-specific focus. These reforms put considerable pressure on policy, but also on research and practice. Rather quick indications of success became increasingly relevant, pushing more or less explicitly towards short-term goal thinking and towards general (non-subject-specific) goals.

In addition, the meanwhile relatively good results in international achievement studies in MINT subjects (although a rich country could aim at more ambitious goals) seemed to decrease the urgent need for subject-specific initiatives like IMST. The increasing competition between universities and colleges, and the increasing ambition of the ministry to transform IMST to colleges (as subordinate agencies of the ministry), and thus to make it more centrally steered, transformed the question of sustainably establishing IMST into a delicate challenge.

The increasing output-steering of universities and university colleges of teacher education influenced the work done in IMST, anyway. The initiative decided in the late 2000s to put more focus on increasing international involvement, in particular through co-applying—together with partner institutions—for European funding, and through publishing in high ranked international journals (e.g., co-editing a special issue on scaling up innovations). This strategic decision was not directly caused by policy, but was surely influenced.

The tendency towards more central steering is accompanied with a tendency to favor a *technical rationality* approach and to underestimate the potential of *reflective rationality*, which, however, decreases the likelihood for finding an adequate balance in the sense of *societal rationality*. If top-down decisions gain importance and negotiations become less relevant, the communities *research* and *practice* lose weight in relation to the *policy* community. An example of this is the implementation of an advisory group for mathematics teaching by the ministry with a leader outside the mathematics education research community, neither consulting a scientific association nor a national centre of this field, in advance.

Overall, the central steering approach by policymakers seems to get overweight, drifting to *technical rationality*, expressing a high belief in implementability of general solutions. On the one hand, the departure from a dominance of input-steering and the increase of more output-steering can be seen as a meaningful step. However, the dominance of output-steering is a kind of throwing out the child with the bathwater. In addition to *input* (which always needs consideration) and *output*, also *processes—*involving research and practice—need to be considered. *Societal rationality* builds on negotiation processes between policy, practice, and research.

The negotiations regarding the future of IMST between representatives of the ministry, of universities, and of university colleges of education are running in a constructive way, however, the above-mentioned contexts do not make it easy to make a prediction which decision will be taken. The suggested new IMST *action plan* aims at building a nationwide professional development system for schools, putting an emphasis on subject-specific didactics, subject-related collaboration among teachers at schools, corresponding school autonomy activities supported by their principals, individual schools’ professional development strategies supported by educational administration and policy, and accompanying research. One essential challenge is that principals develop a kind of “leadership content knowledge” (Cobb & Smith, 2008). This means that subject-related and organizational issues need to be interlinked, building on interdisciplinary reflections, negotiations between communities, visibility and support in society, in short, steps towards *societal rationality*. The example of IMST shows that progress can be made, but it means taking a long breath, having patience, looking beyond borders, and involving a constructive intention by all stakeholders.
